# Complete Genome Sequence of *Xanthomonas citri* subsp. *citri* Strain A^w^12879, a Restricted-Host-Range Citrus Canker-Causing Bacterium

**DOI:** 10.1128/genomeA.00235-13

**Published:** 2013-05-16

**Authors:** Neha Jalan, Dibyendu Kumar, Fahong Yu, Jeffrey B. Jones, James H. Graham, Nian Wang

**Affiliations:** Citrus Research and Education Center, Department of Microbiology and Cell Science, University of Florida, Lake Alfred, Florida, USAa;; Waksman Genomics Core Facility, Rutgers University, Busch Campus, Piscataway, New Jersey, USAb;; ICBR, University of Florida, Gainesville, Florida, USAc;; Department of Plant Pathology, University of Florida, Gainesville, Florida, USAd;; Citrus Research and Education Center, Department of Soil and Water Science, University of Florida, Lake Alfred, Florida, USAe

## Abstract

*Xanthomonas citri* subsp. *citri* causes citrus canker. The Asiatic strain has a broad host range, whereas the Wellington variant has a restricted host range. Here, we present the complete genome of *X. citri* subsp. *citri* strain A^W^12879. This study lays the foundation to further characterize the mechanisms for virulence and host range of *X. citri*.

## GENOME ANNOUNCEMENT

*Xanthomonas* is an important model genus for studying host-microbe interactions, capable of infecting over 390 plant varieties. Among the diseases caused by *Xanthomonas*, citrus canker caused by *X. citri* subsp. *citri* is an important disease that has severe economic impacts on citrus industries worldwide. Canker caused by the *X. citri* subsp. *citri* Asiatic strain is the most widespread and destructive form of citrus canker (1, 2, 3). The Asiatic strain has a broad host range and affects most commercial citrus varieties, including grapefruit, sweet orange, and Mexican lime (4, 5). Two variants of the Asiatic strain, designated A* and A^W^, were identified previously. The A* strain was found in Southeast Asia in the 1990s infecting Mexican lime (5). The A^W^ strain (Wellington strain), isolated from Palm Beach County in southern Florida, was characterized by Schubert et al. in the late 1990s (4, 6). The A^W^ strain was found to be pathogenic to Mexican lime and alemow plants, but not to grapefruit and sweet orange. This strain also causes a hypersensitive reaction (HR) in grapefruit (7). Here, we report the genome sequence of *X. citri* subsp. *citri* strain A^W^12879, isolated from Mexican lime in Florida (4). This sequence adds to the genomic data of previously sequenced citrus canker-causing bacteria, i.e., *X. citri* subsp. *citri* Asiatic strain 306, *X. fuscans* subsp. *aurantifolii* strain B, and *X. fuscans* subsp. *aurantifolii* strain C (8, 9). Our objective is to facilitate comparative genomic studies to characterize the mechanisms for virulence and host range of citrus canker-causing bacteria.

The complete genome of *X. citri* subsp. *citri* strain A^W^12879 was generated by using 454 pyrosequencing and Illumina technology, resulting in average coverage of 24× and 410×, respectively. Newbler 2.0 software was used for *de novo* assembly of 454 sequencing reads into 378 contigs and 17 scaffolds. The 17 scaffolds were further aligned to the BamHI optical map (OpGen technologies) that revealed the orientation of scaffolds and several misassembles. Illumina sequences were assembled using CLC Genomics workbench 5.0 into 1,426 contigs that were used to close gaps between the 454 scaffolds. PCR amplification and Sanger sequencing were used to close the remaining gaps, and Softberry’s FgenesB pipeline was used for finding protein coding sequences (CDS). The predicted CDS were annotated by similarity searches against the NCBI nonredundant (nr) protein database and clusters of orthologous groups (COG) database and curated manually using the JGI GenePRIMP pipeline (10).

The final sequence of *X. citri* subsp. *citri* strain A^W^12879 contains a chromosome (5.32 Mb and 64.71% G+C content) and two circular plasmids, pXcaw19 (18,869 bp and 63.07% G+C content) and pXcaw58 (58,317 bp and 61.85% G+C content). The genome consists of 4,760 annotated CDS, of which 3,457 could be assigned to a COG functional category. The genome harbors 2 rRNA operons and 54 tRNA genes, which were identified with FgenesB and tRNAscan-SE, respectively (11). The plasmid pXcaw19 sequence has no homology with the plasmids of *X. citri* subsp. *citri* Asiatic strain 306, whereas pXcaw58 is only about 35% similar to pXAC64 (8).

### Nucleotide sequence accession numbers.

The complete genome sequence of *X. citri* subsp. *citri* strain A^W^12879 (including one chromosome and two plasmids) has been deposited in GenBank under accession numbers CP003778, CP003779, and CP003780.
